# Haploidentical Stem Cell Transplantation With a Novel Conditioning Regimen in Older Patients: A Prospective Single-Arm Phase 2 Study

**DOI:** 10.3389/fonc.2021.639502

**Published:** 2021-02-26

**Authors:** Yu-Qian Sun, Ting-Ting Han, Yu Wang, Chen-Hua Yan, Feng-Rong Wang, Zhi-Dong Wang, Jun Kong, Yu-Hong Chen, Huan Chen, Wei Han, Yao Chen, Yuan-Yuan Zhang, Xiao-Hui Zhang, Lan-Ping Xu, Kai-Yan Liu, Xiao-Jun Huang

**Affiliations:** ^1^National Clinical Research Center for Treatment of Hematological Disease, Peking University People's Hospital, Peking University Institute of Hematology, Beijing, China; ^2^Beijing Key Laboratory of Hematopoietic Stem Cell Transplantation for the Treatment of Hematological Diseases, Beijing, China; ^3^Peking-Tsinghua Center for Life Sciences, Beijing, China

**Keywords:** haploidentical transplant, elderly, anti-thymocyte globulin, cyclophosphamide, fludarabine

## Abstract

**Objective:** Haploidentical stem cell transplantation (haplo-SCT) has demonstrated encouraging results in younger patients. There is also an increasing need for haplo-SCT in older patients. However, the high risk of treatment-related mortality (TRM) in older patients is still a major concern. We aimed to investigate a novel conditioning regimen (Bu/Flu/Cy/ATG) followed by haplo-SCT in older patients.

**Method:** This prospective, single-arm clinical trial was performed at Peking University Institute of Hematology, China. Patients were enrolled if they were (1) diagnosed with acute leukemia or MDS; (2) without MSD and MUD, and with HID available; and (3) age ≥55 years. The Bu/Flu/Cy/ATG regimen consisted of the following agents: Ara-C (2 g/m^2^/day, injected i.v.) on days-10 and−9; BU (9.6 mg/kg, injected i.v. in 12 doses) on days-8,−7, and−6; Flu (30 mg/m^2^/day, injected i.v.) from day−6 to day−2; Cy (1 g/m^2^/day, injected i.v.) on days−5 and−4; semustine (250 mg/m^2^, orally) on day-3 and antithymocyte globulin (ATG) [2.5 mg/kg/day, rabbit, SangStat (Lyon, France)] on days−5,−4,−3, and−2. The primary endpoint was 1-year TRM.

**Results:** From April 1, 2018 to April 10, 2020, a total of 50 patients were enrolled. All patients achieved neutrophil engraftment with complete donor chimerism. The cumulative incidence of grade 2-4 aGVHD at day-100 was 22.0%. The cumulative incidences of CMV viremia and EBV viremia on day 100 were 68.0 and 20.0%, respectively. The cumulative incidence of TRM at 1-year was 23.3%. and the cumulative incidence of relapse (CIR) at 1 year after transplantation was 16.5%. The overall survival (OS) and leukemia-free survival (LFS) at 1 year were 63.5 and 60.2%, respectively. The outcomes were also comparable with patients who received Bu/Cy/ATG regimen using a propensity score matching method.

**Conclusions:** In conclusion, this study suggested that a novel conditioning regimen followed by haploidentical HSCT might be a promising option for older patients. The study was registered as a clinical trial.

**Clinical Trial Registration:** www.ClinicalTrials.gov, identifier: NCT03412409.

## Introduction

Allogeneic hematopoietic stem cell transplantation (allo-SCT) remains the main and often only curative method for most of the hematological malignancies (1, 2). Historically, allo-SCT was performed from a matched sibling donor (MSD) with a myeloablative conditioning regimen. Allo-SCT was mainly performed in relatively younger patients since the risk of treatment related mortality (TRM) in older patients who received myeloablative conditioning regimen transplant procedures was relatively higher. However, most adult hematological malignancies develop in older individuals, with a median age of ~60–70 years. The prognosis for older patients treated with conventional chemotherapy is very poor, and allo-SCT still provides superior long-term survival compared with conventional chemotherapy in older patients (3). Therefore, there is an increasing need for allo-SCT in older patients and an unmet need to decrease the TRM of allo-SCT to further improve long-term survival.

Among the strategies to reduce TRM, using a regimen with less toxicity is an important option. Since much of the regimen-related toxicity(RRT) (such as the cardiac toxicity, veno-occlusive disease and hemorrhagic cystitis) in myeloablative BuCy regimens is believed to be due to Cy (4), fludarabine has been widely used to reduce the RRT in the setting of MSD and unrelated donor(URD) (5–8). Besides, the development of a reduced intensity conditioning (RIC) regimen has led to improved outcomes of allo-SCT in older patients. These advances have led to the increased use of allo-SCT in older patients (9) in the setting of MSD or URD.

Unfortunately, MSDs are only available for less than one-quarter of patients. Haploidentical donors are a good alternative for those without MSDs (10) with several advantages. First, haploidentical donors (HID) can be available in ~100% of cases and can be used very quickly. Second, the sibling donor for the older patients is usually older and therefore at higher risk of comorbidities and complications during donation. Furthermore, there is evidence that younger haploidentical donors may be superior to older matched sibling donors for older patients. Therefore, haploidentical stem cell transplantation for older patients is a promising option when MSD or URD is unavailable.

Currently, haplo-SCT is mainly performed in younger patients due to the concerns about increased TRM in older patients. Therefore, there is an unmet need to decrease the TRM and further improve the overall survival (OS) of haplo-SCT in older patients. We previously attempted a fludarabine-based regimen (replacing of Cy 3.6 g/m^2^ with fludarabine 150 mg/m^2^) in patients with older age or comorbidities. The OS, leukemia-free survival (LFS), TRM and cumulative incidence of relapse (CIR) were quite encouraging and seemed to be comparable to those receiving the Bu/Cy/ATG regimen. However, the incidence of primary graft failure (PGF) was higher in the Bu/Flu/ATG group than in the Bu/Cy/ATG group (17.6 vs. 3.0%, *P* = 0.04) (11). Several other studies also reported a possible association of Flu with poor engraftment. Therefore, in the current study, we used a novel conditioning regimen with a combination of reduced doses of Cy and fludarabine to ensure engraftment and tolerance. In this prospective study, we demonstrated encouraging results using the Bu/Flu/Cy/ATG regimen for older patients receiving haplo-SCT.

## Patients and Methods

### Study Design

This prospective, single-arm clinical trial was performed at the Peking University Institute of Hematology, China. This study was approved by the ethics committee of Peking University People's Hospital. All patients provided written informed consent before enrollment. The study was registered as a clinical trial (ClinicalTrials.gov: NCT03412409). The inclusion criteria were as follows: patients aged ≥ 55 years and diagnosed with acute leukemia or myelodysplastic syndrome (MDS) with a HID available. The exclusion criteria were (1) with pregnancy; (2) active infection without control; (3) enrollment in other clinical trials within 1 month (4) other contradiction to HSCT; and (5) no informed consent. The exit criteria were (1) refusal to comply with the clinical trial protocol; (2) treatment termination due to the patients' decision; and (3) investigator determination that continuing the clinical trial was inappropriate.

The estimation of sample size was performed using PASS software (version 11.0.7; PASS, NCSS, LLC). The incidence of 1-year TRM was 40% according to our previous study (11). This study proposed reducing the incidence of 1-year TRM from 40 to 20%. The settings included α = 0.05, two sided, β = 0.20, and a sample size of 43 was determined to achieve the statistical power (power = 80%). Meanwhile, considering sample loss due to follow-up, death due to early treatment and other unexpected factors, an additional 10% sample size was required; thus, the final sample size was ~49 patients. No interim analysis was planned.

Patients who received the myeloablative Bu/Cy/ATG conditioning regimen during the current study period were compared with patients who received Bu/ Flu/Cy/ATG in this study. Finally, 100 patients were selected as control group using propensity score matching method with a 1:2 ratio.

### Conditioning Regimens and Other Transplant Procedures

The Bu/Flu/Cy/ATG regimen consisted of the following agents: Ara-C (2 g/m^2^/day, injected i.v.) on days-10 and−9; BU (9.6 mg/kg, injected i.v. in 12 doses) on days-8,−7, and−6; Flu (30 mg/m^2^/day, injected i.v.) from day−6 to day−2; Cy (1,000 mg/m^2^/day, injected i.v.) on days−5 and−4; semustine (250 mg/m^2^, orally) on day-3 and antithymocyte globulin (ATG) [10 mg/kg, rabbit, SangStat (Lyon, France)] on days−5,−4,−3, and -2.

### Donor Selection, Mobilization, and Stem Cell Collection

The donor selection rule was based on previous literature. Patients were eligible for haploidentical HSCT if a MSD or URD was unavailable. All recipients received G-CSF-mobilized bone marrow and peripheral blood-derived stem cells.

### Supportive Therapy

All patients received cyclosporine (CsA), mycophenolate mofetil (MMF) and short-term methotrexate (MTX) for graft-vs.-host disease (GVHD) prophylaxis as previously described (1, 12). The dosage of CsA was 2.5 mg/kg per day, i.v., from day 9 before transplantation until bowel function returned to normal. Then, the patient was switched to oral CsA. MMF was administered orally, at 0.5 g every 12 h, from day 9 before transplantation until hematopoietic recovery after transplantation. The dosage of MTX was 15 mg/m^2^, administered i.v. on day 1, and 10 mg/m^2^ on days 3, 6, and 11 after transplantation. Prophylaxis and treatment of CMV infection after alloHSCT were performed as described previously (13, 14). Ganciclovir was administered during conditioning (through day−2) and acyclovir (400 mg twice a day) was given until the discontinuation of all immunosuppressive agents. Patients also received prophylactic drugs to prevent infection by fungi.

Cytomegalovirus (CMV) and Epstein-Barr virus (EBV) were monitored twice per week via real-time PCR. Hematopoietic chimerism was evaluated by fluorescence *in situ* hybridization (FISH) (for sex-mismatched pairs), or the short tandem repeat technique. The HCT-comorbidity index (HCT-CI) score was evaluated according to the literature (15).

### Study Endpoints

The primary endpoint was 1-year TRM, which was defined as any cause of death other than relapse. The secondary endpoints were engraftment, acute graft-vs.-host disease (aGVHD), chronic GVHD (cGVHD), CMV reactivation, EBV reactivation, relapse, OS, and LFS.

Neutrophil engraftment was defined as the first of 3 consecutive days with an absolute neutrophil count ≥0.5 × 10^9^/l. Platelet engraftment was defined as the first of 7 consecutive days with a platelet count ≥20 × 10^9^/l without transfusion dependence. Complete donor chimerism was defined as the presence of at least 95% donor hematopoietic cells. Primary graft failure (PGF) was defined as the failure to surpass a threshold absolute neutrophil count of 0.5 × 10^9^/L by day 28 after transplantation. Acute graft-vs.-host disease (aGVHD) and chronic GVHD (cGVHD) were graded according to previous criteria (16, 17). OS was defined as the time from the first day of transplantation to death as a result of any cause. Follow-up data for survival were censored when the patient was last verified to be alive. LFS was defined as the time from transplantation to relapse, disease progression, or death, whichever occurred first. Relapse was defined as the reappearance of blasts in the blood, BM (>5%) or any extramedullary site after complete remission (CR).

### Statistical Analysis

Continuous variables are represented as the median, and categorical variables are represented as the percentages. OS, and LFS were estimated using the Kaplan-Meier method. The cumulative incidences of engraftment, GVHD, TRM and relapse were estimated using a competing risks model. Death and relapse without developing GVHD were treated as competing events for GVHD, whereas relapse and TRM were treated as competing events. A *p* < 0.05 for a two-sided test was considered significant. The multivariate Cox proportional model and survival analysis were calculated with SPSS software (SPSS 22.0, Chicago, IL, USA). The cumulative incidence was calculated with R statistical software, version 3.6.0 (R Foundation for Statistical Computing, Vienna, Austria).

Logistic regression was used for the propensity score calculation from the following variables: age, sex, and disease. A 1:2 matching by propensity score was performed by using the nearest neighbor matching method with a caliper width fixed at 0.2. Propensity score matching was carried out using SPSS software (SPSS 22.0, Chicago, IL, USA).

## Results

### Patients and Donor Characteristics

From April 1, 2018 to April 10, 2020, a total of 50 patients were enrolled. In addition, the last follow-up date was May 10, 2020. The median age was 59 (55–64) years old. Among the 50 patients, 5 (10%) had HCT-CI ≥3. Most patients (94%) received a combination of bone marrow and peripheral blood as grafts. Details of the patients, disease, transplant characteristics and donor characteristics are shown in Table 1.

**Table 1:** Patient characteristics.

**Variables**	**Numbers**
**Age, median (range)**	59 (55–64)
**Male (%)**	29 (58%)
**Disease**	
AML	19
CR1/CR2/>=CR3/NR	12/1/2/0
ALL	10
CR1/CR2/>=CR3/NR	7/1/2/0
MDS	21
Low/int-1/int-2/high	0/4/12/5
**Disease risk index (low/high)**	26/24
**HCT-CI**	
HCT-CI 0	24
HCT-CI 1-2	21
HCT-CI>=3	5
**Donor sex, male (%)**	28 (56%)
**Donor age, median (range**)	32 (23–60)
**Donor-recipient ABO blood type**	
Match/	27
major /minor/major+minor mismatch	6/14/3
**Donor-recipient CMV sero status**	
+/+	49
+/–	0
–/+	1
–/–	0
Missing	0
**Donor-recipient EBV sero status**	
+/+	49
+/–	0
–/+	1
–/–	0
Missing	0
**Graft**	0
BM+PB	42
PB	8
MNC	8.63 (5.81–18.19)
CD34	2.30 (0.50–7.39)

*AML, Acute myeloid leukemia; ALL, Acute lymphocyte leukemia; MDS, Myelodysplastic syndromes; CR, Complete remission; HCT-CI, Hematopoietic stem cell transplantation Comorbidity Index; CMV, Cytomegalovirus; EBV, Epstein-Barr virus; BM, bone marrow; PB, peripheral blood; MNC, mononuclear cells*.

### Transplant Outcomes

#### TRM

The cumulative incidence of TRM on day 30, day 100, and 1-year was 0%, 6.4% (95% CI 0–37.5%,) and 23.3% (95% CI, 9.1–37.5%), respectively. There were 14 deaths on study, five primarily due to relapse, two due organ failure and seven due to infection.

#### Engraftment

All the 50 patients achieved neutrophil engraftment with a median time of 13.5 (9–23) days. All patients achieved complete donor chimerism. Forty-two patients (42/50, 84.0%) achieved platelet engraftment with a median time of 13 (8–47) days after HSCT. The cumulative incidence of platelet engraftment at 100 days was 84.2% (95% CI, 73.4–95.0%). Five patients did not achieve platelet engraftment due to a TRM and other 3 patients did not achieve platelet engraftment till the last follow-up.

#### GVHD

**Figure 1:**
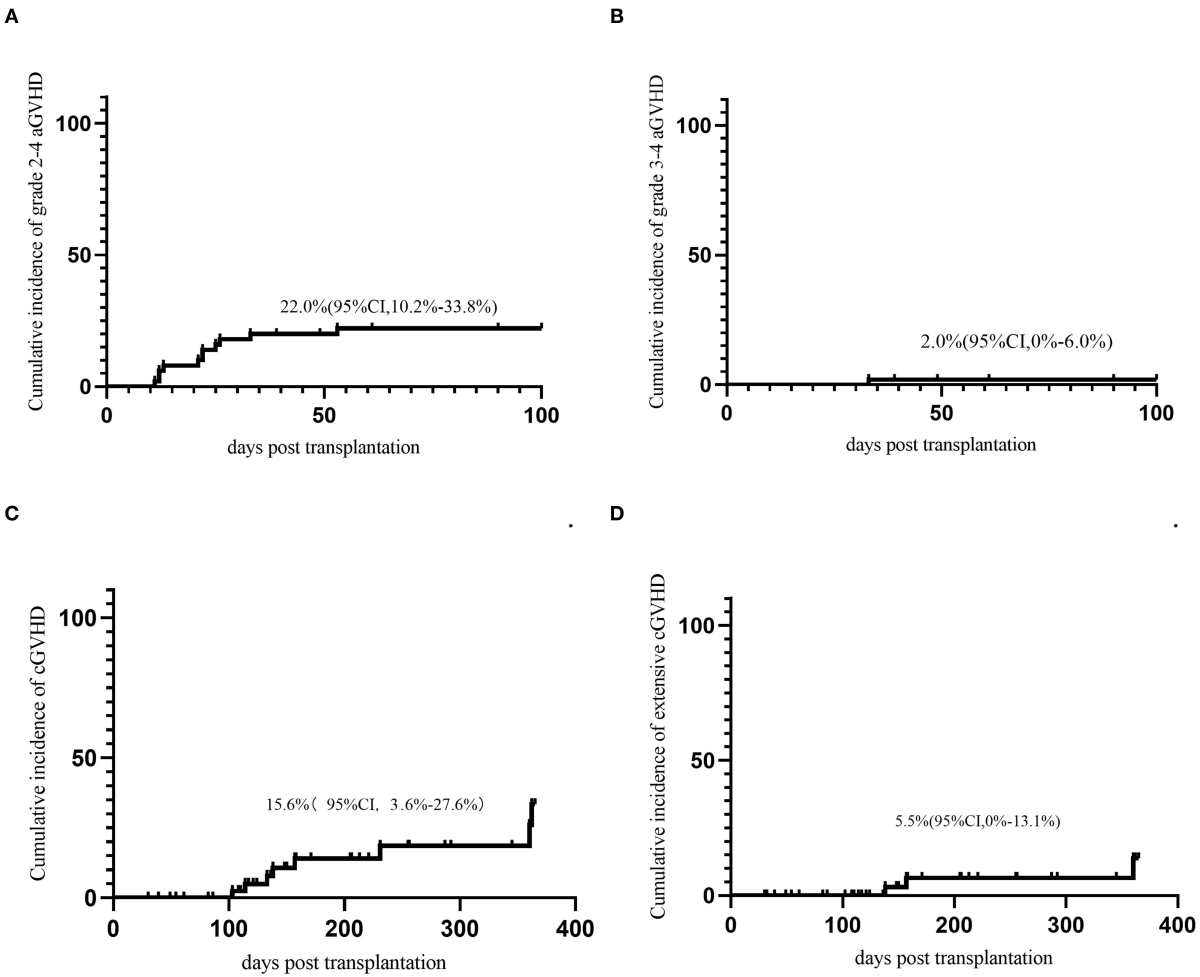
GVHD. **(A)** Acute GVHD grade 2-4; **(B)** Acute GVHD grade 3-4; **(C)** Chronic GVHD; **(D)** extensive chronic GVHD.

Seventeen (30.0%) patients developed aGVHD within 100 days. The median onset time of grade 2-4 aGVHD was 18.4(11–94) days after transplantation. The cumulative incidence of grade 2-4 aGVHD and grade 3-4 aGVD at day-100 was 22.0% (95% CI, 10.2–33.8%) and 2.0% (95% CI 0–6%), respectively (Figures 1A,B). Nine patients developed cGVHD and 4 patients developed extensive cGVHD. The cumulative incidence of cGVHD and extensive cGVHD at 1 year were 15.6% (95% CI, 3.6–27.6%) and 5.5% (95% CI 0–13.1%) (Figures 1C,D).

#### Infection

**Figure 2:**
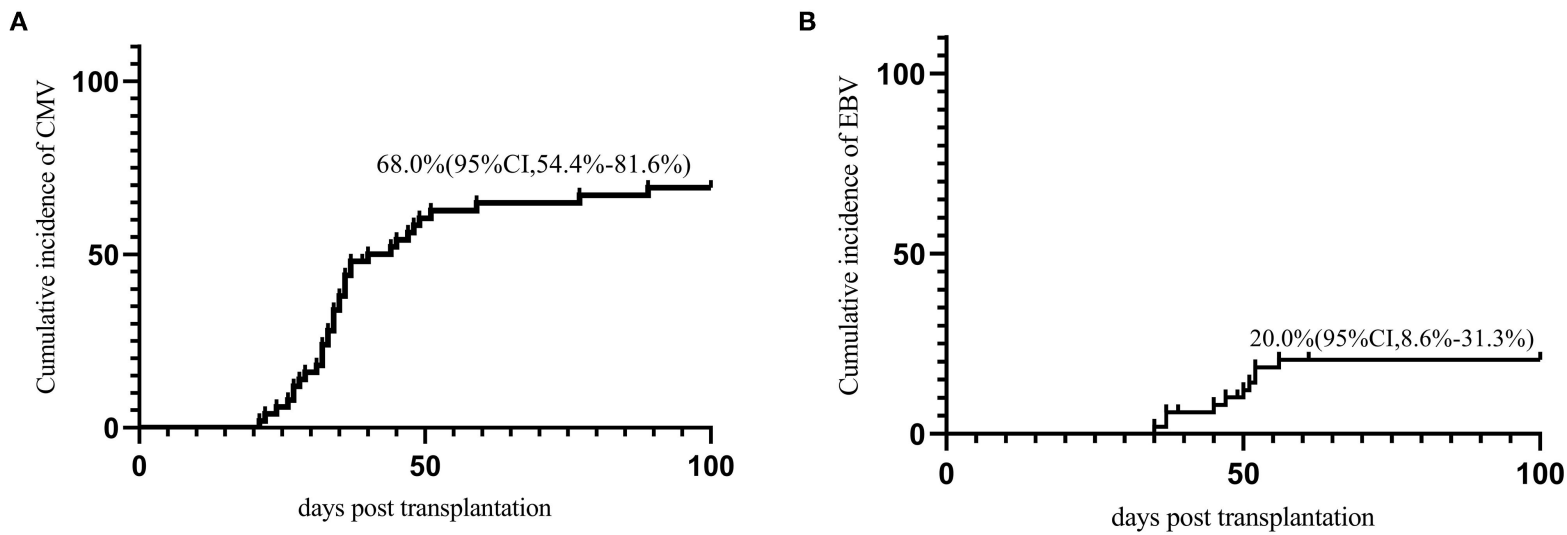
Virus infection **(A)** CMV; **(B)** EBV.

Thirty-four (68.0%) patients developed CMV viremia within 100 days post HSCT. The median onset time of CMV viremia was 34.5 (21–89) days post-HSCT. The cumulative incidence of CMV viremia on day-100 post-HSCT was 68.0% (95% CI, 54.4–81.6%) (Figure 2A). There are two patients developed CMV associated pneumonia. Twelve (24.0%) patients developed EBV reactivation with a median time of 50 (35–101) days post-HSCT. The cumulative incidence of EBV reactivation at day-100 was 20.0% (95% CI, 8.6–31.3%) (Figure 2B). No patients developed PTLD.

#### Relapse and Survival

**Figure 3:**
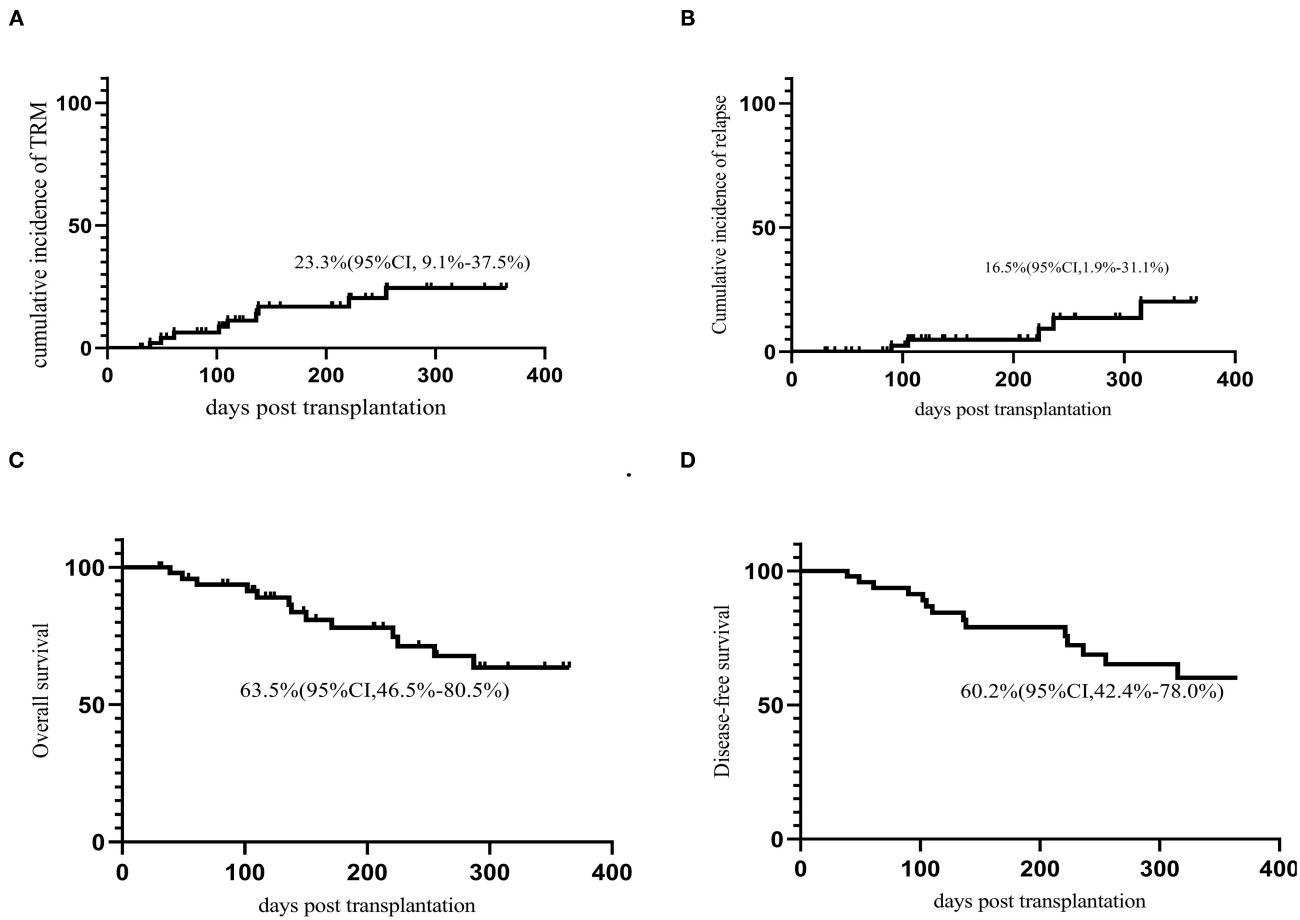
Outcomes **(A)** TRM; **(B)** CIR; **(C)** OS; **(D)** DFS.

The last follow-up date was May 10, 2020. The median follow-up time for survivors was 231.5 (30–754) days after transplantation. Thirty-six patients were still alive at the last follow-up. The cumulative incidence of relapse (CIR) at 1 year after transplantation was 16.5% (95% CI, 1.9–31.1%). The OS and LFS at 1 year were 63.5% (95% CI, 46.5–80.5%) and 60.2% (95% CI, 42.4–78.0%), respectively (Figure 3). No factors were identified to be associated with TRM, LFS, OS, or CIR in multivariate analysis.

#### Comparison With the Bu/Cy/ATG Regimen

The patient characteristics of the two groups of patients are summarized in Supplementary Table 1. The baseline variables were balanced in the two groups except for age, which was younger in the Bu/Cy/ATG group. There were 2 cases of graft failure in the Bu/Cy/ATG group, while there was no graft failure in the Bu/Flu/Cy/ATG group. Compared with the Bu/Cy/ATG group, there were no differences in CMV, EBV, aGVHD, CIR, TRM, LFS, or OS (Supplementary Table 2).

## Discussion

In the present study, we demonstrated that the novel conditioning regimen of Bu/Flu/Cy/ATG followed by haplo-SCT was a feasible option in older patients. This novel haplo-SCT protocol had acceptable TRM with a good profile of engraftment, GVHD, relapse and survival. This encouraging result suggests that haplo-SCT is a potentially promising method for older patients.

Older age historically has been considered a relative contraindication for haplo-SCT, as physicians are often concerned about high TRM in the setting of MAC (18, 19). To address this problem, various reduced-intensity conditioning regimens have emerged. Fludarabine has been widely used to reduce RRT in the setting of MSD and URD (5–8). A fludarabine-based regimen was also used in most reports using haplo-SCT in older patients. Retrospective reports of haplo-SCT using a non-myeloablative regimen (NMA) with either T cell depletion or post-cyclophosphamide (PTCY) have demonstrated that the 2-year TRM is ~27–35%. However, none of the previous retrospective studies reporting haplo-SCT in older patient aimed to evaluate the safety and efficacy of a uniform regimen, and the regimens in the previous retrospective studies were heterogeneous. The incidence of 1-year TRM was 40% according to our previous study (11). With the objective of reducing the TRM, we used a novel MAC regimen consisting of low doses of Cy and fludarabine to reduce the incidence of 1-year TRM from 40 to 20% in our current study. Actuarially, the 1-y TRM was 23% in the present study which seemed to be lower than that in our previous study (Bu/Cy/ATG 29.8–40%) (11). Therefore, it shows that age alone seems to be not a contraindication with current novel method. However, the actual result is discordant with the expected results. For this limitation, we need to expand the samples for further conformation in the future.

Graft failure may be a major concern in situation with reduced intensity of the conditioning regimen. In our previous attempt to complete replacement of Cy 3.6 g/m^2^ by fludarabine 150 mg/m^2^, the incidence of PGF was higher in the Bu/Flu/ATG group than in the Bu/Cy/ATG group (17.6 vs. 3.0%, *P* = 0.04) (11) despite the OS, LFS, TRM and CIR being comparable. Several other studies also reported a possible association of Flu with poor engraftment. In a prospective randomized study comparing BuCy with BuFlu (20), the five graft failures all occurred in the Flu group and the median percentage of recipient hematopoietic chimerism at 4 weeks after transplantation was significantly greater in the BuFlu group (BuCy, 0% [range, 0–7%]; BuFlu, 5.5% [range, 0–94%]; *P* < 0.001). In addition, a group from Nagoya reported that the Flu regimen was a risk factor for donor-type aplasia in children with bone marrow failure diseases (ASH 2012, Abstract 3474; Abstract 959). Another study found that the flu-based conditioning regimen was associated with an increased need for retransplantation compared with the Cy-based regimen (12). Rizzieri et al. reported patients with hematologic malignancies or marrow failure with a 6% incidence of GF and a 10.2% rate of TRM after the Cy (2 g/m^2^) + fludarabine protocol with additional alemtuzumab (16). While transplants in a fludarabine-based conditioning regimen with a high dose of Cy (150 mg/kg) have reportedly been well-engrafted, but with a high of TRM (17). Therefore, it seems that the dose of Cy should be optimized to balance the TRM and engraftment (21). However, the optimal dose of Cy to balance the toxicity and engraftment is still unknown (22). In the current study, we added a reduced dose of Cy (2 g/m^2^) combined with fludarabine to promote engraftment. As expected, our study demonstrated that the addition of Cy to previous Bu/Flu/ATG could promote engraftment compared with a previous report with Bu/Flu/ATG regimen. In accordance with these findings and our experience, this study showed that Cy 2 g/m^2^ may be a balanced dose in combination with Flu + ATG + BU for haplo-HSCT for hematological malignancies, with good engraftment and safety profiles. Further prospective controlled study is warranted to validate the result of this novelconditioning regimen.

Graft-vs.-host disease (GVHD) is the most common complication that affects patients undergone HSCT and it can significantly decrease the quality of life (23–25) and leads to increased late mortality. Acute and chronic GVHD rates appeared to be increased with advancing age (a-GVHD 38 vs. 64% and c-GVHD 45 vs. 54%) in the report from the MD Anderson group (26). Numerous studies have demonstrated that the risk of GVHD is increased with age (27–30). Haploidentical HSCT has comparable or lower GVHD rates than matched-related donor HSCT in patients with PTCY as GVD prophylaxis (31–33). Compared to RIC or NMAC, MAC have a higher incidence of acute and chronic GVHD, which is an important factor contributing to its high TRM (34, 35). However, Scott et al. retrospectively analyzed 1,325 patients aged 18–70 years with AML, ALL, and MDS and received T-cell replete haploidentical HSCT (36). The results showed that there was no difference in grade II-IV aGVHD and c-GVHD according to regimen intensity for older patients. This present study showed a comparable incidence of aGVHD and cGVHD with a novel RTC regimen compared with patients received conventional MAC regimens.

The impact of RIC on TRM in the haplo-SCT setting from retrospective studies is conflicting (18, 36, 37). Although some studies have demonstrated the decreased TRM and reduced toxicity of RIC in older patients or patients with comorbidities, it is also controversial that whether decreased TRM will further translate into improved survival when comparing RIC and MAC regimens. In a propensity score adjusted analysis in haplo-SCT (38), RIC was associated with less TRM but higher relapse and no significant differences in OS, LFS, or GVHD. As for this RTC regimen for older patients, a decreased TRM has been achieved but if the survival was prolonged?

The propensity score matching (PSM) analysis was used to balance the variables affecting the choices of a treatment among different treatment options. So, we conducted a PSM analysis in patients with acute leukemia and MDS to evaluate the effect of the novel conditioning regimen vs. our conventional MAC regimen in our center. The PSM analysis showed that older patients could achieved similar TRM, OS, DFS, and CIR with younger patients. However, whether decreased TRM of this novel regimen could be further translated into improved DFS should be further confirmed in the further prospective RCTs.

In conclusion, our study with a novel conditioning regimen in a homogenous population demonstrated very encouraging results compared with our historical data. It suggested that a novel conditioning regimen of Bu/Flu/Cy/ATG might be a feasible option for older patients receiving unmanipulated haploidentical HSCT. And this novel regimen is worthy of further RCT studies.

## Data Availability Statement

The raw data supporting the conclusions of this article will be made available by the authors, without undue reservation.

## Ethics Statement

The studies involving human participants were reviewed and approved by the ethics committee of Peking University People's Hospital. The patients/participants provided their written informed consent to participate in this study.

## Author Contributions

X-JH conceived and designed the study, reviewed, and edited the manuscript. T-TH collected and analyzed the data. Y-QS assisted in the analysis of the data and wrote the paper. All authors contributed to the revision of the manuscript and approved the final version.

## Conflict of Interest

The authors declare that the research was conducted in the absence of any commercial or financial relationships that could be construed as a potential conflict of interest.
