# Supplementary material for: Haploidentical Stem Cell Transplantation With a Novel Conditioning Regimen in Older Patients: A Prospective Single-Arm Phase 2 Study
Source: Front Oncol. 2021 Feb 26;11:639502. doi: 10.3389/fonc.2021.639502 (PMC7952870; doi:10.3389/fonc.2021.639502)
Supplement: Supplementary file 1 [file Table_1.docx]

**Supplementary Table 1. Patient characteristics**

| **Variables** | **Bu/Flu/Cy/ATG**  **(N=50)** | **Bu/Cy/ATG （N=100）** | **P value** |
| --- | --- | --- | --- |
| Age, median (range) | 59(55-64) | 54(51-61) | <0.001 |
| Male (%) | 29(58%) | 58(58%) | 0.571 |
| Disease |  |  | 0.506 |
| AML | 19 | 47 |  |
| CR1/CR2/>=CR3/NR | 12/1/2/0 | 37/4/6 |  |
| ALL | 10 | 20 |  |
| CR1/CR2/>=CR3/NR | 7/1/2/0 | 18/2/0 |  |
| MDS | 21 | 33 |  |
| Low/int-1/int-2/high | 0/4/12/5 | 11/15/7 |  |
| Disease risk index (low/high) | 26/24 | 42/58 | 0.297 |
| HCT-CI |  |  | 0.009 |
| HCT-CI 0 | 24 | 76 |  |
| HCT-CI 1-2 | 21 | 23 |  |
| HCT-CI>=3 | 5 | 1 |  |
| Donor sex, male (%) | 28(56%) | 70(70%) |  |
| Donor age, median (range) | 32(23-60) | 28(14-56) | <0.001 |
| Donor-recipient ABO bloodtype |  |  | 0.396 |
| Match/ | 27 | 59 |  |
| major /minor/major+minor mismatch | 6/14/3 | 19/18/4 |  |
| Donor-recipient CMV sero status |  |  | 0.713 |
| +/+ | 49 | 93 |  |
| +/- | 0 | 0 |  |
| -/+ | 1 | 3 |  |
| -/- | 0 | 1 |  |
| Missing | 0 | 3 |  |
| Donor-recipient EBV sero status |  |  | 0.690 |
| +/+ | 49 | 95 |  |
| +/- | 0 | 0 |  |
| -/+ | 1 | 1 |  |
| -/- | 0 | 1 |  |
| Missing | 0 | 3 |  |
| Graft | 0 | 0 | <0.001 |
| BM+PB | 42 | 100 |  |
| PB | 8 | 0 |  |
| MNC | 8.63(5.81-18.19) | 8.27(5.79-13.30) | 0.224 |
| CD34 | 2.30(0.50-7.39) | 2.65(0.88-7.12) | 0.246 |
