# Supplementary material for: Haploidentical Stem Cell Transplantation With a Novel Conditioning Regimen in Older Patients: A Prospective Single-Arm Phase 2 Study
Source: Front Oncol. 2021 Feb 26;11:639502. doi: 10.3389/fonc.2021.639502 (PMC7952870; doi:10.3389/fonc.2021.639502)
Supplement: Supplementary file 2 [file Table_2.docx]

**Supplementary Table 2. Comparison of outcomes between the Bu/Flu/Cy/ATG and the Bu/Cy/ATG group**

| Endpoint | Bu/Flu/Cy/ATG | Bu/Cy/ATG | P value |
| --- | --- | --- | --- |
| ANC time | 13.5 (9-23) | 13(9-23) | 0.679 |
| PLT time | 13 (8-47) | 16(7-117) | 0.529 |
| 100d-CMV | 68.0% (54.4-81.6%) | 61.0% (51.2%-70.8%) | 0.176 |
| 100d-EBV | 20.0% (8.6%-31.3%) | 10.0% (4.0%-16.0%) | 0.090 |
| 100d-aGVHD | 22.0% (10.2%-33.8%) | 23.4% (14.8%-32.0%) | 0.950 |
| 1-y-cGVHD | 15.6% (3.6%-27.6%) | 20.6% (12.4%-28.8%) | 0.765 |
| 1-y-TRM | 23.3% (9.1%-37.5%) | 20.1% (11.9%-28.3%) | 0.953 |
| 1-yCIR | 16.5% (9.0%-31.1%) | 17.2% (9.6%-31.4%) | 0.534 |
| 1-y LFS | 60.2% (42.4%-78.0%) | 62.6% (52.8%-72.4%) | 0.734 |
| 1-y OS | 63.5% (46.5%-80.5%) | 67.6% (58.2%-77.0%) | 0.954 |
